# Successful treatment of recurrent small bowel adenocarcinoma by cytoreductive surgery and chemotherapy: a case report and review of the literature

**DOI:** 10.1186/1752-1947-4-213

**Published:** 2010-07-17

**Authors:** Tomoki Yamano, Eiichi Morii, Isao Arai, Toshiaki Takada, Katsuyuki Aozasa

**Affiliations:** 1 Department of Surgery, Kawachi General Hospital, 1-31 Yokomakura, Higashiosaka, Osaka 578-0954, Japan; 2 Department of Pathology, Osaka University Graduate School of Medicine, Suita, Osaka 565-0871, Japan

## Abstract

**Introduction:**

Small bowel adenocarcinoma is a rare malignancy associated with a poor prognosis and there is little evidence of effective treatment. Recurrent small bowel adenocarcinoma is an intractable disease for which there is little information available regarding its treatment by palliative therapy. We present a case of recurrent small bowel adenocarcinoma successfully treated by cytoreductive surgery and palliative chemotherapy.

**Case presentation:**

We report the case of a 72-year-old Japanese female who developed a peritoneal metastasis from recurrent small bowel adenocarcinoma after curative resection and adjuvant chemotherapy with S-1 and polysaccharide K. She underwent cytoreductive surgery followed by chemotherapy with folinic acid/fluorouracil/oxaliplatin and folinic acid/fluorouracil/irinotecan with polysaccharide K. Subsequently, no sign of a recurrence was observed 42 months after the second operation.

**Conclusion:**

To the best of our knowledge, this is the first case report of the successful treatment of peritoneal metastasis from small bowel adenocarcinoma by cytoreductive surgery and combination chemotherapy (folinic acid/fluorouracil/oxaliplatin and folinic acid/fluorouracil/irinotecan with polysaccharide K).

## Introduction

Primary malignancies of the small bowel represent only 2.4% of all gastrointestinal malignancies [1]. Adenocarcinoma is the most common malignancy of the small bowel, comprising about one-third of all small bowel malignancies [2]. The most frequent location for small bowel adenocarcinoma (SBA) is the duodenum (52%-55%), followed by the jejunum (18%-25%) and the ileum (13%), and not otherwise specified (10%-14%) [2,3]. Non-specific symptoms and the lack of useful diagnostic methods results in a delayed diagnosis of SBA. The ratio of SBA diagnosed at stage I, stage II, stage III and stage IV has been reported to be 4%-12%, 20%-27%, 26%-39% and 32%-35%, respectively [2,3]. The five-year survival rate is 26%-30%, with a median survival of 20 months [2,3]. Although curative resection is the most important prognostic factor, 67% of patients with SBA receive curative resection [2,3]. Even after curative resection, 39% develop recurrence [3]. The usefulness of adjuvant chemotherapy after curative resection or palliative chemotherapy for advanced or recurrent SBA remains unconfirmed because of the absence of a randomized control trial (RCT) for SBA [4,5]. Only a few controlled clinical studies for SBA treatment have been reported [6,7] but the effectiveness of chemotherapy for advanced SBA has been shown by retrospective studies [8]. Aggressive surgical intervention seems to be effective for some cases of advanced or recurrent SBA [9,10]. We report on a patient who had peritoneal metastasis from recurrent SBA after curative surgery and adjuvant chemotherapy. Cytoreductive surgery and palliative chemotherapy were employed for this patient.

## Case presentation

A 70-year-old Japanese female was referred to our hospital for the evaluation of a small bowel obstruction. She presented with nausea and loss of weight two months before she consulted her family doctor. An upper gastrointestinal barium study and stoppage of ileus tube movement demonstrated a tumor in the jejunum near the ligament of Treitz. Abdominal computed tomography (CT) revealed a huge uterine myoma. During surgery, we found the jejunal tumor located 20 cm away from the ligament of Treitz. We performed an enterectomy, including the regional mesentery and a hysterectomy.

The jejunal tumor was a moderately differentiated adenocarcinoma, penetrating the small bowel wall. None of the 10 resected lymph nodes were positive for metastasis. The tumor was diagnosed as stage II according to the American Joint Committee on Cancer staging system. Although the usefulness of adjuvant chemotherapy for SBA has not been confirmed due to the lack of RCTs, recurrence after curative resection of SBA is very high [3,4,8]. We therefore decided to administer S-1 (Taiho Pharmaceutical, Tokyo, Japan; 80 mg/day, 2-week administration with 2-week interval) and polysaccharide K (PSK) for 12 months as adjuvant treatment. S-1 has been confirmed to be an effective reagent for gastric cancer after curative surgical resection [11]. PSK has been confirmed as an effective adjuvant for gastric cancer as well as colorectal cancer (CRC) after curative resection [12,13].

Nine months after the surgery, the serum tumor marker carcinoembryonic antigen (CEA) was elevated to 6.1 ng/mL (normal, <5.0 ng/mL). Although body CT and contrast magnetic resonance imaging (MRI) displayed no sign of recurrence, the CEA level was elevated to 8.9 ng/mL the following month. A careful evaluation of her medical history disclosed a hematuria. We consulted a urologist and a bladder tumor was discovered using a cystoscope. Positron emission tomography (PET) revealed fludeoxyglucose accumulation in the ascending colon, sigmoid colon, rectum and bladder. The histology of the bladder tumor resected transurethrally revealed a moderately differentiated adenocarcinoma invading from outside the bladder. A colonoscopy showed tumors in the sigmoid colon and rectum but not in the ascending colon. A histological analysis revealed that these tumors were well-differentiated adenocarcinomas, indicating peritoneal dissemination of SBA. We performed a laparotomy to confirm peritoneal dissemination and to reduce the number of disseminated tumors as cytoreductive surgery has been shown to be useful in some cases of peritoneal carcinomatosis from SBA [9,10].

**Figure 1:**
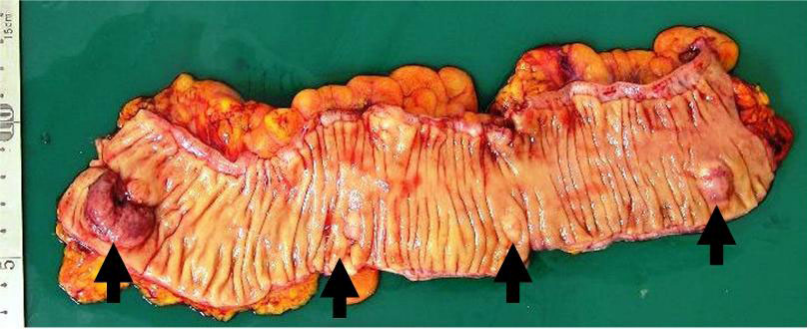
**Resected colon specimen**. Resected colon specimen revealed three tumors in the sigmoid colon and one tumor in the rectum. The features indicated that these were submucosal tumors.

**Figure 2:**
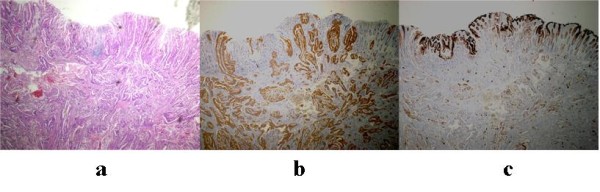
**Histological study of the rectal tumor.** (a) Hematoxylin-eosin staining of the rectal tumor. Hematoxylin-eosin staining showed a moderately differentiated adenocarcinoma in the rectal tumor. The mucosa was not affected by the tumor, thus indicating that this tumor did not originate from the rectal mucosa. (b) CK7 staining of the rectal tumor. The rectal tumor was strongly positive for cytokeratin (CK) 7, whereas the normal mucosa was negative for CK7. (c) CK20 staining of the rectal tumor. The rectal tumor was negative for CK20, whereas the normal mucosa was positive for CK20.

During surgery, we found two sigmoid colon tumors, one rectal tumor and one bladder tumor, identical to the preoperative diagnosis (Figure 1). Besides these tumors, nodules in the larger omentum and intestinal mesentery were recognized. The nodule in the larger omentum was confirmed to be an adenocarcinoma by intraoperative rapid pathological diagnosis. We resected these tumors, including a total cystectomy, rectosigmoidectomy and omentectomy. An immunohistochemical examination of cytokeratin (CK) 7 and CK20 exhibited CK7 (+)/CK20 (-) tumors in the primary and metastatic lesions. This result differed from the profile of CK7/CK20 in CRC and was compatible with the profile of CK7/CK20 in SBA reported previously [14]. Peritoneal carcinomatosis of the colon and bladder was confirmed by histological analyses (Figures 1 and 2).

The effectiveness of palliative chemotherapy for advanced SBA has been described in a number of retrospective studies [8,15-17]. We therefore used folinic acid/fluorouracil/oxaliplatin (FOLFOX) and folinic acid/fluorouracil/irinotecan (FOLFIRI) as these regimens are useful for patients with advanced or recurrent CRC. After the second operation, the patient received 12 cycles of FOLFOX with PSK until the peripheral neuropathy became severe. After this regimen, five cycles of FOLFIRI with PSK were continued until no sign of recurrence was confirmed by PET, CT and laboratory data 12 months after the second operation. We combined PSK with FOLFOX/FOLFIRI because we expected PSK to enhance the efficacy of these chemotherapies. The patient survived with no sign of recurrence 42 months after the second operation.

## Discussion

SBA is rarer than CRC or gastric cancer [1]. This rarity is associated with a poor prognosis, lack of standard treatment and an absence of RCTs [5,18]. In the largest retrospective study, chemotherapy with 5-FU and a platinum compound was more effective than other chemotherapy combinations [15]. A recent prospective study showed the usefulness of capecitabine and oxaliplatin for advanced SBA [7]. SBA treatment is usually extrapolated from CRC or gastric cancer treatment. We extrapolated the first treatment from that of gastric cancer and the second treatment from that of CRC. Although the first treatment involving S-1 and PSK was ineffective, the second treatment using cytoreductive surgery and chemotherapy with FOLFOX/FOLFIRI plus PSK was very effective. Irinotecan has been used for advanced SBA and has shown effectiveness in those cases [8,16,17].

Cytoreductive surgery including parietal peritonectomy with intraperitoneal hyperthermic chemotherapy has demonstrated the effectiveness of peritoneal dissemination for abdominal malignancies including advanced SBA [9,10,18]. Levine *et al. *showed that the primary tumor site, performance status, resection status and development of complications predicted outcomes [19]. Although the extent of our cytoreductive surgery was less than that of the complete cytoreductive surgery by Marchettini *et al. *and Jacks *et al*., we resected all visible tumors. This patient survived for an extended period with good performance status (0), good resection status and no complications. The pattern of peritoneal carcinomatosis from SBA resembles that from CRC than from gastric cancer. An extrapolation of CRC treatment to SBA treatment seems necessary before confirming a standard treatment.

## Conclusions

Cytoreductive surgery and chemotherapy with FOLFOX/FOLFIRI plus PSK seemed useful for recurrent small bowel adenocarcinoma.

## Abbreviations

CEA: carcinoembryonic antigen; CK: cytokeratin; CRC: colorectal cancer; CT: computed tomography; FOLFOX: folinic acid/fluorouracil/oxaliplatin; FOLFIRI: folinic acid/fluorouracil/irinotecan; MR: magnetic resonance; PET: positon emission tomography; PSK: polysaccharide K; RCT: randomized control trial; SBA: small bowel adenocarcinoma.

## Consent

Written informed consent was obtained from the patient for publication of this case report and the accompanying images. A copy of the written consent is available for review by the Editor-in-Chief of this journal.

## Competing interests

The authors declare that they have no competing interests.

## Authors' contributions

TY was the primary physician and surgeon, conceived the original study, organized and analyzed the data and prepared the draft of the manuscript. EM and KA were the pathologists and carried out the histological examinations. AI and TT carried out surgery, evaluated laboratory and imaging data and assisted with manuscript editing. All authors read and approved the final manuscript.
